# Vector‐borne disease and its relationship to hematologic abnormalities and microalbuminuria in retired racing and show‐bred greyhounds

**DOI:** 10.1111/jvim.16477

**Published:** 2022-07-11

**Authors:** Linda Kidd, Helen Hamilton, Lisa Stine, Barbara Qurollo, Edward B. Breitschwerdt

**Affiliations:** ^1^ Western University of Health Sciences College of Veterinary Medicine Pomona California USA; ^2^ Retired Wilsonville Oregon USA; ^3^ Independent Contractor Scio Oregon USA; ^4^ Vector‐Borne Disease Diagnostic Laboratory North Carolina State University College of Veterinary Medicine Raleigh North Carolina USA

**Keywords:** *Babesia*, *Bartonella*, *Ehrlichia* thrombocytopenia, hemotropic *Mycoplasma*, leukopenia, proteinuria

## Abstract

**Background:**

Reference intervals for platelets and white blood cell (WBCs) counts are lower in greyhounds than other breeds. Proteinuria is common. Vector‐borne diseases (VBD) cause thrombocytopenia, leukopenia, and proteinuria. Racing greyhounds are commonly exposed to vectors that carry multiple organisms capable of chronically infecting clinically healthy dogs.

**Hypothesis/Objectives:**

Vector‐borne disease prevalence is higher in retired racing greyhounds than in show‐bred greyhounds. Occult infection contributes to breed‐related laboratory abnormalities.

**Animals:**

Thirty National Greyhound Association (NGA) retired racing and 28 American Kennel Club (AKC) show‐bred greyhounds.

**Methods:**

Peripheral blood was tested for *Anaplasma*, *Babesia*, *Bartonella*, *Ehrlichia*, hemotropic *Mycoplasma*, and *Rickettsia* species using PCR. Antibodies to *Anaplasma*, *Babesia*, *Bartonella*, *Ehrlichia*, and *Rickettsia* species and *Borrelia burgdorferi* were detected using immunofluorescence and ELISA assays. Complete blood counts, semiquantitative platelet estimates, and microalbuminuria concentration were determined.

**Results:**

Seven of 30 NGA and 1/28 AKC greyhounds tested positive for ≥1 VBD (*P* = .05). More positive tests were documented in NGA (10/630) than in AKC dogs (1/588; *P* = .02). Exposure to *Bartonella* species (3/30), *Babesia vogeli* (2/30), *Ehrlichia canis* (1/30), and infection with *Mycoplasma hemocanis* (3/30) occurred in NGA dogs. Platelet counts or estimates were >170 000/μL. White blood cell counts <4000/μL (4/28 AKC; 5/30 NGA, *P* > .99; 1/8 VBD positive; 8/51 VBD negative, *P* = .99) and microalbuminuria (10/21 AKC; 5/26 NGA, *P* = .06; 1/8 VBD positive; 14/25 VBD negative, *P* = .41) were not associated with VBD.

**Conclusions and Clinical Importance:**

The prevalence of thrombocytopenia and *B. vogeli* exposure was lower than previously documented. Larger studies investigating the health impact of multiple VBD organisms are warranted.

**Abbreviations**
ACVIM: American College of Veterinary Internal Medicine
AKC: American Kennel Club
EDTA: ethylenediaminetetraacetic acid
IFA: immunofluorescence assay
NGA: National Greyhound Association
PCR: polymerase chain reaction
VBD: vector‐borne disease
WBC: white blood cell

## 1 INTRODUCTION

Greyhounds are popular companion animals in the United States. Many are retired racing greyhounds registered with the National Greyhound Association (NGA), (https://www.ngagreyhounds.com/Home). Show‐bred greyhounds registered with the American Kennel Club (AKC) have pedigrees that certify they have not raced (http://www.greyhound-data.com). Therefore, these 2 groups have distinct genetic and environmental backgrounds.

Greyhounds have several unique clinicopathologic and physiologic traits.^1^, ^2^, ^3^, ^4^, ^5^, ^6^, ^7^, ^8^, ^9^, ^10^, ^11^, ^12^, ^13^, ^14^ Platelet and leukocyte counts are lower than in other breeds, and proteinuria is common.^1^, ^3^, ^7^, ^8^, ^9^, ^10^, ^11^, ^13^, ^14^ Thrombocytopenia, leukopenia, and proteinuria are associated with vector‐borne diseases (VBD).^15^, ^16^, ^17^, ^18^, ^19^, ^20^, ^21^, ^22^, ^23^, ^24^, ^25^, ^26^, ^27^, ^28^ Many VBD organisms chronically infect otherwise apparently healthy dogs.^29^, ^30^, ^31^, ^32^, ^33^ Because these clinicopathologic findings are considered breed‐related, VBD screening may not be pursued in thrombocytopenic, leukopenic, or proteinuric greyhounds as recommended for other breeds.^33^, ^34^, ^35^ For example, investigation of thrombocytopenia is not recommended in greyhounds unless platelet counts are <100 000/μL.^3^, ^10^

Racing greyhounds are commonly exposed to *Rhipicephalus sanguineus*.^36^, ^37^ This tick is adapted to kennel environments.^38^ It commonly causes infestations in the southern and mid‐Atlantic United States and Mexico, where greyhound racing occurs (http://www.greyhound-data.com; http://www.fastfriends.org/).^36^, ^37^, ^39^, ^40^, ^41^ *Rhipicephalus sanguineus* is an established or suspected vector for *Ehrlichia canis*, *Babesia vogeli* (formerly *B. canis*), spotted fever group *Rickettsia*, *Bartonella* species, *Anaplasma platys*, hemotropic *Mycoplasma* and *Babesia conradae*.^42^, ^43^, ^44^, ^45^, ^46^, ^47^, ^48^, ^49^, ^50^, ^51^, ^52^, ^53^, ^54^ Hemotropic *Mycoplasma* and small *Babesia* species also are hypothesized to be transmitted vertically or by biting, which might contribute to their transmission in kennels.^25^, ^55^, ^56^, ^57^, ^58^ The distribution of *Amblyomma americanum* also includes the southern and mid‐Atlantic United States. It is the vector for *Ehrlichia chaffeensis* and *Ehrlichia ewingii*, which cause chronic subclinical infections in dogs.^29^, ^30^ In previous serosurveys, approximately 50% of greyhounds in the United States were *B. vogeli* seroreactive.^36^, ^37^ Infection with this organism is more common in greyhounds than other breeds.^58^ *Ehrlichia canis* seroprevalence has ranged between 0.4% and 12% in greyhounds.^37^, ^59^ Infection with *B. conradae* recently was documented in coyote hunting greyhounds and greyhound mixes in California and Oklahoma.^25^, ^60^ The prevalence of *A. platys*, *Bartonella* spp., *E. ewingii*, *E. chaffeensis* and hemotropic *Mycoplasma* in racing greyhounds has not been reported.

Greyhounds of unspecified lineage, racing greyhounds, retired racing greyhounds and greyhounds from “hunting kennels” have been used to investigate breed‐associated clinicopathologic differences.^7^, ^8^, ^9^, ^10^, ^11^, ^12^, ^13^ Vector‐borne disease screening was mentioned in 1 study, and was limited to serologic testing for *Dirofilaria immitis* and, for some dogs, *B. canis* (*vogeli*) and *E. canis*.^7^ Therefore, occult VBD may have been present in outwardly healthy dogs used to establish breed differences. We hypothesized that VBD is more prevalent in retired racing greyhounds than in show‐bred greyounds, that organisms in addition to *B. vogeli* and *E. canis* are common in retired racing greyhounds, and that occult infection contributes to “breed‐related” thrombocytopenia, leukopenia and microalbuminuria.

Our primary objectives were to:

1. Compare the prevalence of exposure to, or infection with, *Anaplasma phagocytophilum*, *A. platys*, *B. vogeli*, *B. gibsoni*, *B. conradae*, *Bartonella henselae*, *Bartonella vinsonii* subsp. *berkhoffii*, *Bartonella koehlerae*, *E. canis*, *E. chaffeensis*, *E. ewingii*, and spotted fever group (SFG) *Rickettsia* in retired racing greyhounds and show‐bred greyhounds.
2. Determine if the prevalence of thrombocyoptenia, leukopenia, and microalbuminuria, and the magnitude of platelet counts, leukocyte counts and microalbuminuria differs between retired‐racing and show‐bred greyhounds.
3. Determine if exposure to, or infection with, VBD agents is associated with thrombocytopenia, leukopenia, and microalbuminuria in greyhounds.

## 2 METHODS

Twenty‐eight show‐bred AKC registered greyhounds and 30 NGA registered retired racing greyhounds were enrolled in the study. Informed signed consent from the owner was required for participation. This study was approved by the Western University of Health Sciences IACUC committee, protocol # R16IACUC043. Dogs were considered clinically healthy if there was no history of coughing, sneezing, vomiting, diarrhea, polyuria or polydipsia and if physical examination abnormalities were limited to dental disease, a heart murmur or minor cutaneous abnormalities. Physical examinations were performed by American College of Veterinary Internal Medicine boarded internists (LK and HH) on the day of phlebotomy. Blood was collected by single atraumatic venipuncture of the jugular vein by an experienced technician (LS) and immediately transferred into 2 EDTA and 2 red top vacutainer blood collection tubes. Two blood smears were made immediately at the time of phlebotomy. A voided urine sample was collected into clean specimen cups. Serum was separated by low‐speed centrifugation at the time of phlebotomy. Anticoagulated blood, serum and urine samples were submitted on the day of collection to a commercial diagnostic laboratory (Antech Diagnostics) for CBC, serum biochemistry, urinalysis and microalbuminuria quantitation. Semiquantitative platelet estimates were made from the 2 blood smears by an experienced cytologist at Stat Veterinary Laboratory, San Diego, CA who was unaware of the dog's VBD or racing status. The number of platelets/μL were determined by calculating the average number of platelets in 10 high power fields (hpf; 100 Χ oil immersion) at the monolayer and multiplying by 15 000. The average count for the 2 slides was used for estimated platelet count calculations. Any platelet clumps that were present were not included in estimates so that numbers were potentially under, rather than over estimated.

The second set of EDTA‐anticoagulated blood and serum samples was shipped overnight to the Vector Borne Disease Diagnostic Laboratory at North Carolina State University. Samples were batched and stored at −80°C until analysis. The DNA was extracted from EDTA anti‐coagulated whole blood using QIAsymphony^SP^ (Qiagen, Hilden, Germany) with QIAsymphony® DNA Mini Kit (192) (Qiagen). Polymerase chain reaction was performed to detect *Ehrlichia* spp., *Anaplasma* spp., *Babesia canis*, *B. gibsoni*, *B. conradae*, SFG *Rickettsia*, *Bartonella* spp., and hemotropic *Mycoplasma* species DNA.^24^, ^61^, ^62^, ^63^, ^64^, ^65^ Samples positive at the genus level were speciated using additional PCR assays or amplicon DNA sequencing as previously described by our laboratory.^63^, ^64^, ^65^ Immunofluorescence assay (IFA) was performed to detect antibodies to *B. vogeli*, *B. gibsoni*, *Bartonella henselae*, *B. vinsonii* subsp. *berkhoffii*, *B. koehlerae*, *Rickettsia rickettsii*, and *E. canis*. Antibody responses to *E. canis* (NCSU CO‐89 Jake strain), *B. vinsonii* subsp*. berkhoffii* (NCSU CO‐93), *B. henselae* Houston‐1 (NCSU 93FO‐23) *B. koehlerae* (NCSU FO‐1‐09), *B. gibsoni*, and *B. vogeli* were tested by IFA as previously described by our laboratory.^66^ Seroreactive samples are defined as having endpoint titers ≥1 : 64 using a scale of 1 : 16 to 1 : 8192. An ELISA was used to detect antibodies to *E. canis*, *E. ewingii*, *A. phagocytophilum*, *A. platys*, and *Borrelia burgdorferi* using the SNAP® 4DX®PLUS test kit according to the manufacturer's instructions.

### 2.1 Statistical analysis

Power calculations for the outcomes of detecting differences in prevalence of VBD, thrombocytopenia and microalbuminuria between groups were calculated using epitools.ausvet.com.au (power = 0.8, *α* = 0.05). Based on previous studies, the seroprevalence of *B. vogeli* in NGA dogs and AKC dogs was assumed to be 50% and 0%, respectively.^36^, ^37^ To detect a difference in VBD prevalence between groups the required sample size was estimated to be n = 15 in each group. Approximately 50% of greyhounds are thrombocytopenic according to most reference intervals established for other breeds (<170 000 platelets/μL).^7^, ^9^, ^10^ Assuming 50% of NGA and 0% of AKC greyhounds are thrombocytopenic, the required sample size again was estimated to be n = 15 in each group. Fifty‐three percent of urine samples collected from clinically healthy retired racing greyhounds by cystocentesis are positive for microalbuminuria whereas up to 15% of voided urine samples from clinically healthy dogs of other breeds exhibit microalbuminuria.^13^, ^67^ We assumed the prevalence of microabluminuria to be at least 53% in voided urine of NGA and 15% in AKC greyhounds, requiring a sample size of n = 29 in each group.

For statistical comparisons, thrombocytopenia was defined as <170 000 platelets/μL, leukopenia as a total white blood cell (WBC) count of <4000/μL, and microalbuminuria as urine albumin concentration >2.5 mg/dL, as established by the reference laboratory (Antech Diagnostics). Reported values of >30 mg/dL were considered to be equivalent to 30 mg/dL for statistical comparisons. Samples with bacteriuria, pyuria, or macroscopic hematuria were excluded from the microalbuminuria analyses. A dog was considered VBD positive if a positive serologic or PCR result for VBD testing was documented.

All statistical analyses were performed using statistical software (GraphPad Prism version 7.00 for Windows, GraphPad Software, La Jolla, California and graphpad.com/quickcalcs). Fisher's exact test was performed for contingency analysis of categorical variables. Continuous variables were tested for normality using D'Agostino and Pearson normality tests. For nonparametric data, median and range are reported, and for normally distributed data, mean and standard deviation are reported. Mann‐Whitney tests were performed when at least 1 group in a comparison lacked a Gaussian distribution. *T*‐tests were performed when data in groups being compared were normally distributed. *P* < .05 was considered statistically significant.

## 3 RESULTS

Signalment: Fifty‐eight dogs were enrolled in the study, 28 AKC show bred greyhounds and 30 NGA retired racing greyhounds. There were 17 spayed females and 13 neutered males in the NGA group and 3 spayed females, 13 intact females, 4 neutered males, and 8 intact males in the AKC group.

Complete blood count, serum biochemistry, and VBD testing was performed for all dogs. Urine samples were obtained from 52/58 dogs. Five samples with bacteriuria were excluded from the microalbuminuria analyses. No samples had pyuria or macroscopic hematuria.

Eight of the 58 dogs (14%) had evidence of exposure to or active infection with a VBD agent. The NGA retired racing greyhounds had more exposure to (seroreactive), or infection with (PCR positive), ≥1 VBD (7/30) than did AKC show‐bred greyhounds (1/28), but the difference in overall prevalence between the 2 groups was not significant (*P* = .05). Post hoc testing showed more VBD agents were detected in NGA than in AKC dogs (NGA, n = 10 positive tests/630 tests; AKC, n = 1 positive test/588 test; *P* = .02). Coexposures and infections were common. In the retired racing greyhounds, exposure to (seroreactivity to) *Bartonella* species and infection (PCR+) with *M. haemocanis* were most common, being documented in 3 dogs each (10%). Two dogs were seroreactive to *B. vogeli* (1 : 64 and 1 : 128) and 1 dog was *E. canis* seroreactive (1 : 256). Coexposure occurred in 3 of these dogs, 1 was seroreactive to both *B. henselae* (1 : 64) and *B. vinsonii* subsp. *berkhoffii* (1 : 64); 1 was seroreactive to both *E. canis* (1 : 256) and *B. koehlerae* (1 : 64) and 1 was seroreactive to both to *B. vogeli* (1 : 128) and *B. koehlerae* (1 : 128). One AKC show‐bred greyhound had a low (1 : 64) positive titer to *R. rickettsii*. Notably, IFA titers to all organisms were of low magnitude (Supplementary Table 1).

Data sets without a Gaussian distribution included total leukocyte count for AKC; AKC total neutrophil count, NGA total monocyte count, AKC and NGA total eosinophil count; NGA quantitative platelet count; magnitude of microalbuminuria AKC, NGA, VBD, and no VBD. All other data sets were normally distributed.

**FIGURE 1:**
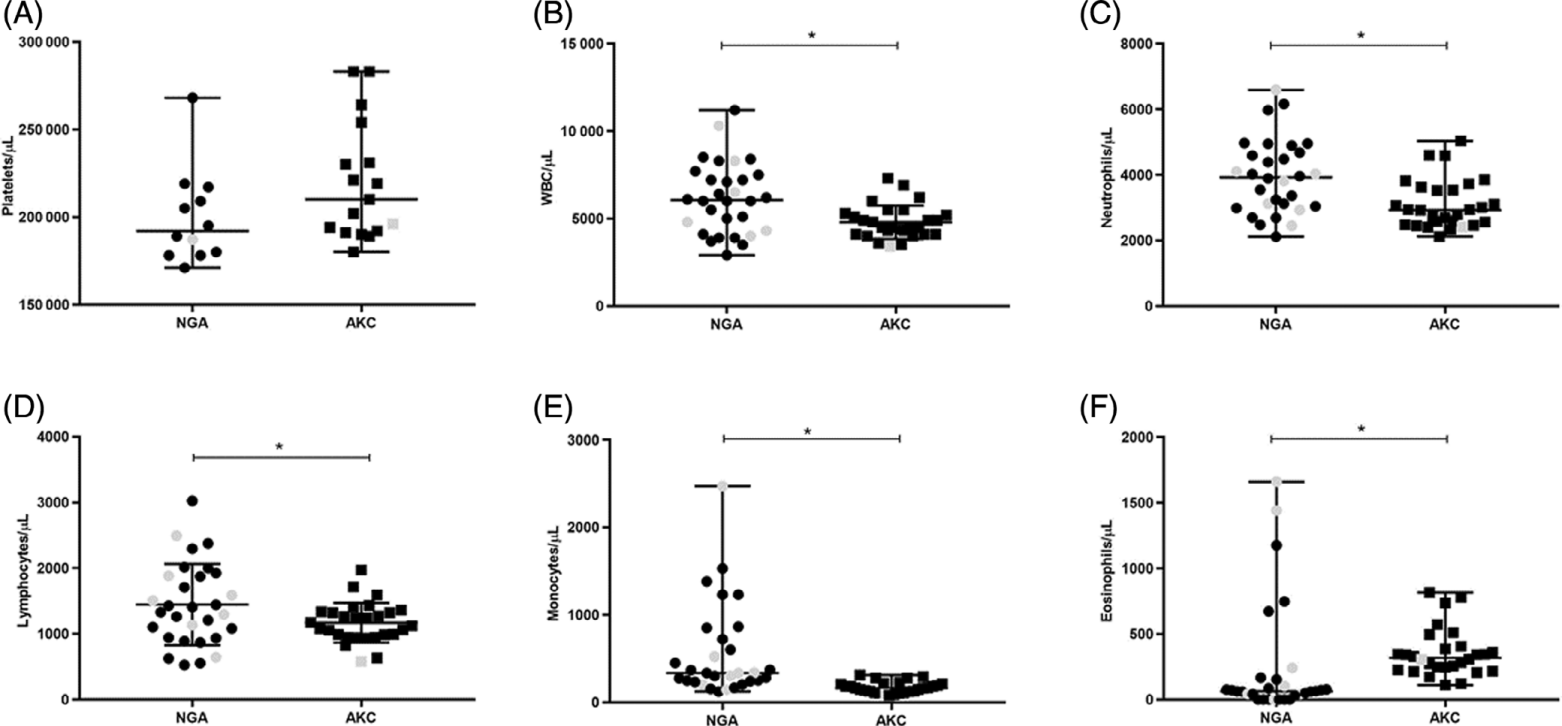
Platelet and WBC counts in retired racing (NGA, n = 30 unless otherwise indicated) and show‐bred (AKC, n = 28 unless otherwise indicated) greyhounds are displayed. *P < .05; gray fill = VBD positive; black fill = VBD negative. (A) Quantitative platelet counts in n = 12 NGA (median, 192 000; range, 171 000‐268 000/μL) and n = 17 AKC, (median, 210 000; range, 180 000‐283 000/μL) dogs (*P* = .05). (B) Total WBC counts in NGA (median, 6050/μL; range, 2900‐11 200/μL) and AKC (median, 4500/μL; range, 3400‐7300/μL) dogs (*P* = .01). (C) Total neutrophil count in NGA (median, 3924/μL; range, 2117‐6592/μL) and AKC (median, 2933/μL; range, 2120‐5037/μL) dogs (*P < *.01). (D) Total lymphocyte count in NGA (mean, 1445 ± 619/μL) and AKC (mean, 1168 ± 303.6 μL) dogs. (*P* = .04). (E) Total monocyte count in NGA (median, 332.5/μL; range, 123‐2472/μL) and AKC (median, 162; range, 80‐310/μL) dogs. (*P* ≤ .001). (F) Total eosinophil count in NGA (median, 63/μL; range, 0‐1660) and AKC (median, 318/μL; range, 110‐816/μL) dogs (*P* < .001)

No dog in either group was thrombocytopenic (<170 000 platelets/μL) according to quantitative platelet counts and semiquantitative platelet estimation performed as described above. Platelet clumping was reported in 29/58 samples, and these samples were excluded from quantitative platelet analyses. Based upon quantitative platelet counts, none of the remaining 29 dogs were thrombocytopenic (Figure 1A). Two greyhounds with positive VBD test results had quantitative platelet counts without clumping (1 NGA; platelet count, 187 000/μL and 1 AKC; platelet count, 196 000/μL). Semiquantitative platelet estimates were calculated for 57/58 dogs. No difference (*P* = .65) was found in semiquantitative platelet estimates between AKC (n = 28; mean, 308 200 ± 53 520/μL) and NGA (n = 29, mean, 314 100 ± 47 280/μL dogs. No difference was found in semiquantitative platelet estimates between dogs with exposure to, or infection with, VBD organisms (n = 8; mean, 303 100 ± 53 060/μL) and dogs with negative VBD test results (n = 49; mean, 312 500 ± 50 030/μL; *P* = .63).

Based upon WBC counts of ≤4000/μL, 9 dogs were leukopenic. Leukopenia was not more common in the 8 dogs with VBD exposure or infection (1/8) compared to those without VBD exposure or infection (8/50; *P* = .99). Leukopenia was not more common in AKC (4/28) than NGA dogs (5/30; *P* > .99). Total WBC, neutrophil, lymphocyte and monocyte counts were lower and eosinophil counts were higher in AKC dogs compared to NGA dogs (Figure 1B‐F).

**FIGURE 2:**
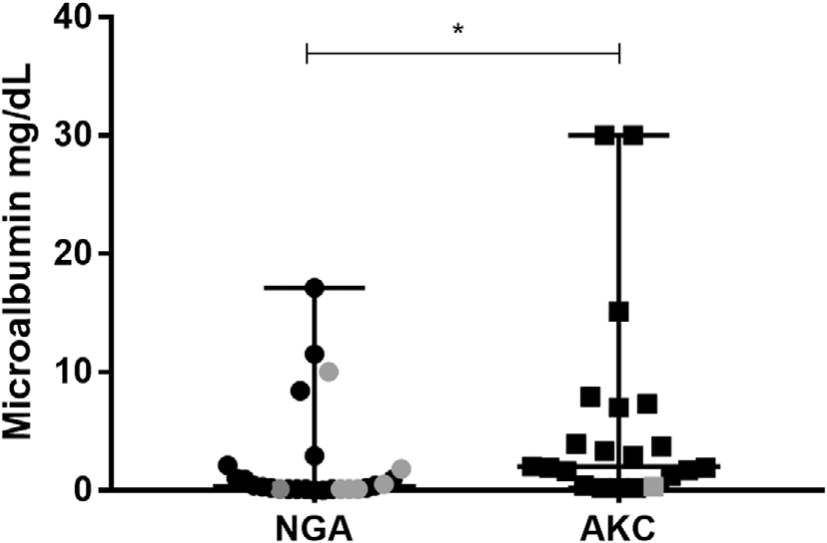
Magnitude of microalbuminuria in retired racing (NGA, n = 26) and show‐bred (AKC, n = 21) greyhounds. Median and range (error bars) are displayed. Gray fill = VBD positive, black fill = VBD negative. One dog with an indicated value of 30 mg/dL had a reported value of >30 mg/dL. *P = .005

More AKC show‐bred greyhounds (10/21) had microalbuminuria than did retired racers (5/26), but the difference was not significant (*P* = .06). The magnitude of microalbuminuria was higher in AKC (median, 2.0; range, 0.2‐ >30 mg/dL) than in NGA (median, 0.4; range, 0.0‐17.1) greyhounds (*P* < .01; Figure 2). Microalbuminuria was not more common in VBD positive (1/8) dogs compared to VBD negative (14/39) dogs (*P* = .41), and the magnitude of proteinuria was not different between VBD positive (median, 0.2 mg/dL; range, 0.1‐10) and VBD negative (median, 1.6 mg/dL; range, 0‐ >30 mg/dL; *P* = .07; Figure 2).

## 4 DISCUSSION

The overall prevalence of at least 1 positive test for VBD in an individual retired racing greyhound was not significantly higher in retired racing greyhounds compared to show‐bred greyhounds. However, the retired racing greyhounds tested positive to more VBD agents than did show‐bred greyhounds, and coinfection or coexposure to ≥1 agent was common.

Previous studies have suggested that racing greyhounds are at increased risk for exposure to or infection with *B. canis* (*vogeli*) and possibly *E. canis* because of risk of exposure to *R. sanguineus* in racing kennels.^36^, ^37^, ^58^, ^59^ Interestingly, in our study, the seroprevalence of *B. vogeli* was much lower that previously reported. In a 1983 study of 8 racing farms in the southeastern United States, the mean seroprevalence for *B. canis* (*vogeli*) was 52%,^37^ and in a 1992 study of greyhounds in Florida, 46% were seroreactive to *B. canis* (*vogeli*).^36^ In contrast, we found the seroprevalence for *B. vogeli* was 6.67% of retired racing greyhounds. This observation is likely because of increased use of effective acaricides by racing greyhound breeders and trainers and increased screening and treatment for *B. vogeli*, by greyhound rescue agencies in recent years. Interestingly, the medical records for 2 of the dogs in our study contained *B. vogeli* screening results for 15 additional retired racing greyhounds before adoption. Nine of those 15 dogs (60%) were *B. vogeli* seroreactive, which is more consistent with the seroprevalence in previous reports. This finding suggests that rescue agencies may aggressively screen or treat for the organism before adoption.

In addition to *B. vogeli* and *E. canis*, *R. sanguineus* also is a known or suspected carrier of *Mycoplasma* spp., and *Bartonella* spp.^43^, ^44^, ^45^, ^46^, ^47^, ^48^, ^49^, ^50^, ^51^, ^52^, ^53^, ^54^ Ours is the first study to specifically test for and identify *Bartonella* (10%) exposure and hemotropic *Mycoplasma* species (10%) infection in retired racing greyhounds. *Bartonella* IFA serology using the 3 antigens in our study has specificity ≥97%, but the sensitivity can be as low as 50%.^68^ Thus, *Bartonella* spp. exposure may represent an underestimate for dogs in our study. Exposure to *Bartonella* spp. and infection with hemotropic *Mycoplasma* recently have been documented in kennels of coyote hunting greyhounds.^25^, ^60^ In addition to transmission by arthropod vectors, the authors hypothesized that some agents may be transmitted vertically or through biting.^24^, ^68^, ^69^ The species and diversity of organisms and high prevalence of coinfection in retired racing greyhounds may reflect cotransmission of multiple pathogens by *R. sanguineus* or simultaneous exposure to multiple vectors or other means of transmission. Taken together, these studies illustrate the importance of comprehensive screening for VBD in dogs with these occupational and breed exposure risks.^24^, ^69^

Unexpectedly, thrombocytopenia was not found in either the retired racing greyhounds or the show‐bred greyhounds. Previous studies to establish normal reference ranges for platelets in greyhounds have led to the conclusion that greyhounds have lower platelet counts than other breeds, and that platelet counts between 100 000 and 170 000/μL should not be considered abnormal in this breed.^3^, ^7^, ^9^, ^10^, ^11^ However, a high rate of exposure to *R. sanguineus*, other ticks, and potentially fleas in some geographic locales may have contributed to the low platelet counts historically reported in greyhounds as a breed. Indeed, the retired racing greyhounds in our study had serologic evidence of exposure to *Bartonella*, *Babesia*, and *Ehrlichia* species, all of which are associated with thrombocytopenia. However, it is important to note that no dog tested PCR positive for these organisms, and titers were low. Therefore, a lack of active infection may explain the uniformly normal platelet counts observed in our study. Although 3 dogs were actively infected with (PCR+) *M. haemocanis*, infection with this organism is not usually associated with thrombocytopenia or other laboratory abnormalities in healthy dogs.^43^, ^70^, ^71^ Therefore, it is not surprising that even dogs in the VBD group in our study were not thrombocytopenic. Larger, contemporary studies re‐exploring reference intervals in greyhounds comprehensively screened for VBD agents are warranted.

Like thrombocytopenia, leukopenia and leukocytosis are variably associated with VBD. Some studies have shown that WBC counts are lower in greyhound dogs than in other breeds.^3^, ^8^, ^11^ We explored whether occult VBD may account for leukopenia in some greyhounds. We did not find that leukopenia occurred more commonly in greyhounds testing positive for VBD compared to those testing negative.

Surprisingly, we found that total WBC counts (including neutrophils, lymphocytes, and monocytes) were lower and eosinophils were higher in the show‐bred dogs compared to the retired racing dogs. In addition to infection, other factors such as stress, age, sex, and neuter status can affect WBC counts in dogs.^72^, ^73^, ^74^ Whether these or other factors account for differences requires further investigation.

Proteinuria is also common in both greyhound dogs and in dogs with VBD.^13^, ^16^, ^17^, ^18^, ^19^, ^20^, ^21^, ^22^, ^25^, ^34^ In our study, microalbuminuria was common in both retired racing greyhounds and in show‐bred greyhounds and was not significantly more frequent in dogs with evidence of VBD exposure. This finding may be explained by a lack of active infection with VBD agents such as *Babesia* and *Ehrlichia* spp. that cause microalbuminuria. In addition, other mechanisms such as breed‐related vascular dysfunction likely contribute to proteinuria in this breed.^2^ There were more intact males in the AKC than in the NGA group which, in addition to genetic differences, may have contributed to the higher magnitude of microalbuminuria in the AKC group.^75^

One limitation of our study was that platelet clumping precluded the use of quantitative platelet counts for some analyses. Although venipuncture was atraumatic and samples were kept refrigerated during the collection process, platelet clumping was very common. Greyhound platelets are more reactive than those of other breeds, potentially contributing to the propensity for clumping despite atraumatic venipuncture.^3^, ^10^ Notably, the prevalence of clumping seen in the greyhounds in our study was similar to a previous study of greyhound platelets.^10^ In addition, a delay of several hours occurred from when blood was placed into EDTA tubes and when automated platelet counts were performed at the laboratory because of the field conditions of our study. It is possible that the semiquantitative platelet estimates overestimated platelet counts. However, the formula used in our study would tend to underestimate rather than overestimate the platelet count.^76^ In addition, previous reports have shown the magnitude of thrombocytopenia is <150 000/μL in 5% to 53% of retired racing greyhounds.^7^, ^9^, ^10^ Decreases of this magnitude should be detected using blood smear‐based estimates.

Another limitation was the unexpectedly low prevalence of exposure to VBD agents compared to older studies of *B. vogeli* exposure in retired racing greyhounds.^36^, ^37^ This, coupled with the normal platelet counts in all dogs, precluded us from determining if VBD has any role in contributing to lower platelet counts historically considered normal for the breed, and our ability to detect whether a difference was present in overall prevalence of VBD between groups with our chosen sample size. Despite this limitation, our results suggest reference intervals for greyhounds should be further assessed by larger scale studies using comprehensive screening for VBD as exclusionary criteria. In addition, like other breeds, retired racing greyhounds with clinically relevant thrombocytopenia should be screened for vector‐borne and other diseases.

## 5 CONCLUSIONS

We found retired racing greyhounds are exposed to a variety of VBD agents. In addition to *B. vogeli* and *E. canis*, exposure to *Bartonella* species and infection with *Mycoplasma hemocanis* was documented. The seroprevalence of *B. vogeli* was much lower than previously reported in the 1980's and 1990's, likely because of improved acaracide prevention by breeders and trainers, as well as increased screening and antimicrobial treatment by rescue agencies. We did not document active infection with any VBD organism associated with thrombocytopenia, but we also did not detect thrombocytopenia in any dog. Exposure to, or infection with, VBD agents was not associated with leukopenia or proteinuria. Because PCR was negative, and titers were of low magnitude, a lack of active infection with VBD organisms that cause these clinicopathologic abnormalities may explain these findings. Exposure risk should be considered when assessing the cost‐benefit ratio of VBD testing in greyhounds with compatible clinical and laboratory findings also considered to be breed‐related. Larger, sequential studies that include comprehensive screening for multiple VBD agents using both serology and PCR may help clarify the role of occult VBD in the some of the breed‐related clinical and laboratory abnormalities in greyhounds.

## CONFLICT OF INTEREST DECLARATION

Dr Kidd serves as Associate Editor for the Journal of Veterinary Internal Medicine. She was not involved in review of this manuscript. Dr Kidd is a key opinion leader for IDEXX Diagnostic Laboratories and is a paid speaker for IDEXX Diagnostic Laboratories and Zoetis Animal Health. Antech Diagnostics provided diagnostics for this study at a reduced research fee.

Dr Breitschwerdt is a key opinion leader and paid speaker for IDEXX Diagnostic Laboratories. IDEXX Laboratories provided the SNAP 4DX+ kits used in this study. He is also the Director Intracellular Pathogens Research Laboratory and the Codirector, Vector‐Borne Disease Diagnostic Laboratory at North Carolina State University College of Veterinary Medicine.

Dr Qurollo is an Associate Research Professor at NC State‐CVM. IDEXX Laboratories, Inc funds a portion of her salary. She is also Co‐Director of the Vector‐Borne Disease Diagnostic Laboratory at North Carolina State University College of Veterinary Medicine.

Dr Hamilton and Ms Stine have no conflict of interest to declare.

## OFF‐LABEL ANTIMICROBIAL DECLARATION

Authors declare no off‐label use of antimicrobials.

## INSTITUTIONAL ANIMAL CARE AND USE COMMITTEE (IACUC) OR OTHER APPROVAL DECLARATION

Informed signed consent from the owner was required for participation. This study was approved by the Western University of Health Sciences IACUC committee, protocol # R16IACUC043.

## HUMAN ETHICS APPROVAL DECLARATION

Authors declare human ethics approval was not needed for this study.

## Supporting information


**Supplementary Table 1** Supporting Information.
